# Structure of a 10–23 Deoxyribozyme Exhibiting a Homodimer Conformation

**DOI:** 10.21203/rs.3.rs-2252941/v1

**Published:** 2023-05-31

**Authors:** Evan Cramer, Sarah Starcovic, Rebekah Avey, Ali Kaya, Aaron Robart

**Affiliations:** West Virginia University; West Virginia University; West Virginia University; West Virginia University

**Keywords:** Deoxyribozyme, DNAzyme, RNA, DNA, structure

## Abstract

Deoxyribozymes (DNAzymes) are *in vitro* evolved DNA sequences capable of catalyzing chemical reactions. The RNA cleaving 10–23 DNAzyme was the first DNAzyme to be evolved and possesses clinical and biotechnical applications as a biosensor and a knockdown agent. DNAzymes do not require the recruitment of other components to cleave RNA and can turnover, thus they have a distinct advantage over other knockdown methods (siRNA, CRISPR, morpholinos). Despite this, a lack of structural and mechanistic information has hindered the optimization and application of the 10–23 DNAzyme. Here, we report a 2.7 Å crystal structure of the RNA cleaving 10–23 DNAzyme in a homodimer conformation. Although proper coordination of the DNAzyme to substrate is observed along with intriguing patterns of bound magnesium ions, the dimer conformation likely does not capture the true catalytic form of the 10–23 DNAzyme.

## Introduction

Deoxyribozymes (DNAzymes) are short (15–40 nt) single-stranded DNA molecules that catalyze chemical reactions^1^. These catalytic DNAs are not found in nature, rather they are selected from a random oligonucleotide library and enriched for a desired activity, allowing for the precise control of both the desired reaction type and condition^2^. DNAzymes generally consist of two substrate binding arms flanking a central catalytic core. The flanking binding arms can be altered both in length and in sequence identity, allowing high substrate specificity and limiting off-target effects. DNAzymes are also capable of enzymatic turnover and do not require the recruitment of other components to function. These qualities give DNAzymes the potential to be an efficient and specific knockdown agent that could be applied in a clinical or biotechnical setting.

DNAzymes have been artificially selected to catalyze numerous reactions such as RNA cleavage, RNA/DNA ligation, DNA base excision, diels-alder, thymine dimer resolution, and numerous other activities^3,4,5,6^. RNA cleavage is the most common reaction performed by DNAzymes. Spurred by the discovery that base modification of DNAzymes improves both catalytic efficiency and stability *in vivo*, DNAzymes as therapeutics has grown in popularity over the last decade^7,8,9^. In recent years, there have been clinical trials of the 10–23 DNAzyme for use in cancer and asthma treatment by targeting the reduction of protein expression through catalyzed mRNA cleavage^10,11^. Unfortunately, DNAzymes currently lack the desired efficiency for *in vivo* use and optimization is currently hindered by the lack of structural and mechanistic information^12^. To this point, two previous catalytically relevant crystal structures of DNAzymes have been reported, one of the RNA-ligating 9DB1 DNAzyme and of the RNA-cleaving 8–17 DNAzyme^13,14^. The RNA-cleaving 10–23 DNAzyme has been of high interest since its discovery 37 years ago due to utilization of the physiologically available cofactors such as magnesium^15^. There have been previous attempts to determine the structure of the 10–23 DNAzyme^16,17^. Recently, NMR and molecular dynamics yielded predicted structures at high resolution^18^. These structures, while providing insight into a potential monomeric 10–23 DNAzyme conformation could not inform metal coordination. Additional structural information is necessary to support these previous structures, inform magnesium ion coordination patterns, and to provide additional mechanistic insight. To address this need, we have obtained a 2.7 Å crystal structure of the 10–23 DNAzyme-substrate complex captured in precatalytic conditions.

## Results and Discussion

### *The 10–23 DNAzyme forms a dimer* in crystallo

**Figure 1:**
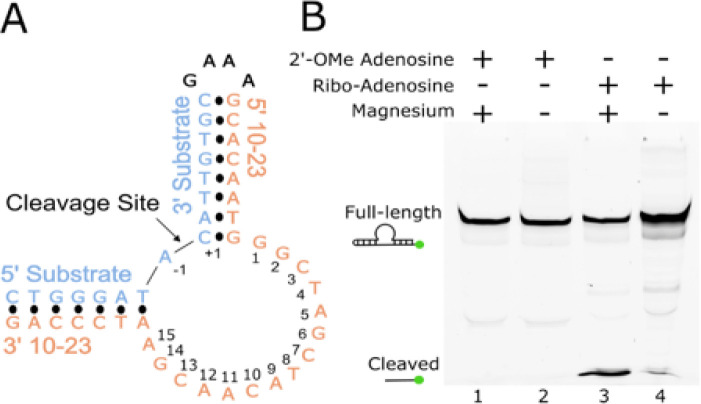
10–23 DNAzyme crystallization construct and activity. (A) Secondary structure of the 10–23 crystallization construct. (B) Activity of the 10–23 crystallization construct showcasing inhibition of activity with the 2′-OMe substitution at the cleavage site (green dots indicate position of the FAM label).

**Figure 2:**
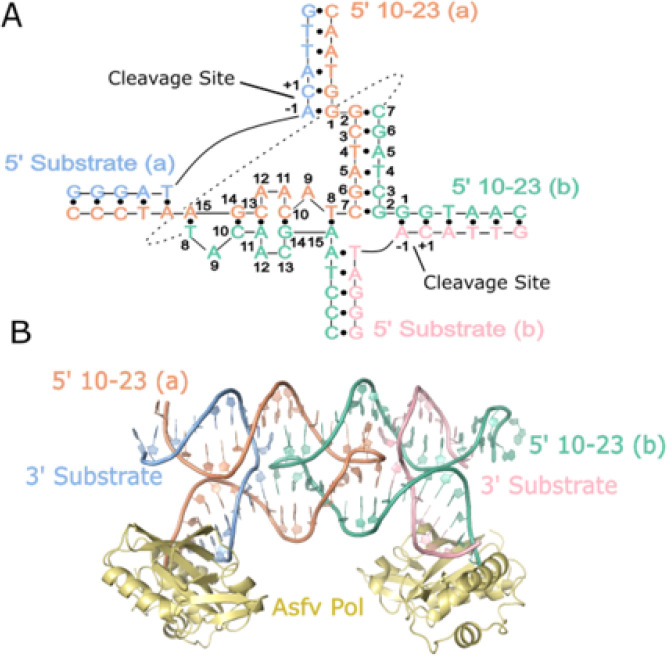
The crystal structure of the 10–23 DNAzyme forms a homodimer. (A) Secondary structure depicting base-pair interactions between the DNAzyme catalytic cores that coordinate the homodimer conformation. (B) Overview of the crystal structure with crystallization chaperone Asfv PolX.

A dimer conformation has been observed previously in 10–23 DNAzyme crystal structures with these structures being interpreted to not possess proper alignment of the cleavage site to the catalytic core^16,17^. This dimerization phenomena is largely due to the presence of a palindromic sequence within the catalytic core of the 10–23 DNAzyme. Through this interaction the 10–23 DNAzyme, when in complex with a physically separate ssDNA or ssRNA substrate, yields a four-way junction conformation that was concluded to not be mechanistically informative (Supplemental Fig. 2)^16,17^. To combat the crystallization forces contributing to artifact formation, the substrate and DNAzyme were placed on the same continuous ssDNA strand with a snapback GAAA tetraloop to allow for alignment of the scissile phosphate within the DNAzyme catalytic core (Fig. 1A). This oligonucleotide contains a single ribonucleotide position (−1A), that uses its 2′-OH as the attacking nucleophile to cleave the adjacent 3′ phosphoribose backbone position. Self-cleavage activity of this construct was shown by *in vitro* activity assays, slight activity without the presence of magnesium is attributed to background hydrolysis of the ribo-adenosine position (Fig. 1B). The activity of this construct is low compared to other variants of the 10–23 DNAzyme due to the use of the A-C substrate cleavage site which has been found to be the least active cleavage site nucleotide combination^19^. Despite its lower activity, mfold predicted this substrate to be the most uniformly folded, a quality that is essential for successful crystallography^20^. In addition, the design of the DNAzyme crystallization construct is expected to possess low activity because the construct, by nature, cannot turnover due to product inhibition and lack of free excess substrate. To capture a precatalytic 10–23 DNAzyme structure, the 2′-OH position of the RNA nucleophile was modified by 2′-O-methyl (2′-OMe) substitution which was shown to attenuate activity *in vitro*. After folding, the construct forms a single blunt dsDNA end which was bound to the African Swine Fever Polymerase X (Asfv polX) as recently described for the 8–17 DNAzyme^21^. The 10–23 homodimer possesses proper coordination of DNAzyme to substrate unlike previous structures, however, still does not capture the primary catalytic form of the 10–23 DNAzyme. The dimer is mediated through two regions of antiparallel base pairing within the catalytic core. The region (G2-C7) is palindromic and is responsible for most of the base pair interactions that occur within the dimer. The second region of interaction (T8-A15) doesn’t possess continuous base pairing like the palindromic region but features a base stacking interaction between A11 and A12 of both catalytic cores (Fig. 2A).

### *The 10–23 Homodimer exists as a minor population* in vitro

**Figure 3:**
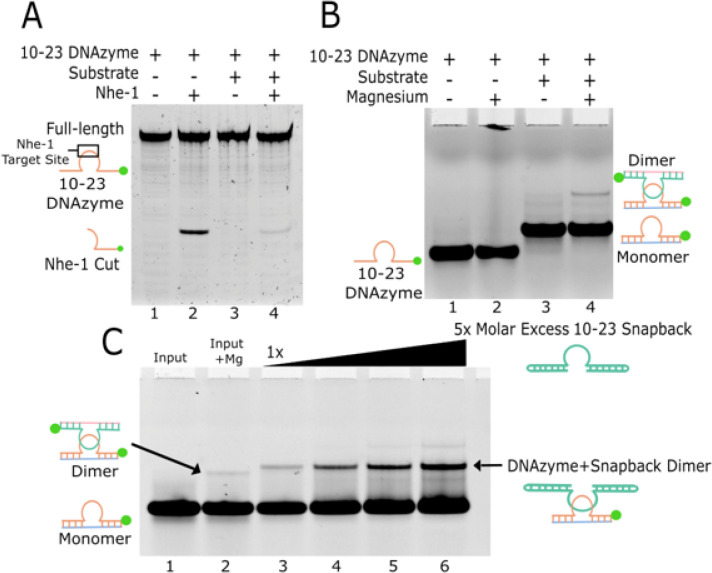
*In vitro* evidence of 10–23 dimerization. (A) Restriction enzyme cleavage of the palindromic sequence withing the 10–23 DNAzyme catalytic core. (B) EMSA revealing dimer formation in the presence of substrate and magnesium. (C) EMSA with a heavier unlabeled snapback DNAzyme which shifts and enriches the dimer band (green dots indicate position of the FAM label).

*In vitro* experiments were performed to determine the presence of the dimer in solution. Conveniently, the palindromic sequence within the 10–23 DNAzyme matches the consensus site for the restriction enzyme Nhe I, which will specifically cleave double stranded DNA sequences. Overnight incubation of the DNAzyme with Nhe I produced the expected restriction enzyme reaction products, demonstrating the presence of the dimer in solution (Fig. 3A). In the DNAzyme only digestion, the cleavage product accounted for ~ 25% of the sample. Addition of the substrate reduced the cleavage product to ~ 3% (Fig. 3B lanes 2,4). The discrepancy between these two values can be explained by the addition of the substrate strand causing steric hinderance that limits Nhe I activity.

The extent of dimer presence in typical DNAzyme reaction conditions was determined through electrophoretic mobility shift assays (EMSAs) using fluorescently labeled 10–23 DNAzyme. Unlabeled 2’-OMe substrate was utilized to trap the DNAzyme in its precatalytic state consistent with the crystal structure. Interestingly, the formation of the 10–23 dimer was only observed in the presence of both substrate and magnesium, occurring in a concentration dependent manner (Fig. 3B lane 4). However, even in these optimal reaction conditions, the dimer only accounted for ~ 5% of the DNAzyme population. The identity of the dimer was confirmed by titrating an unlabeled DNAzyme with extended binding arms possessing a snapback loop mimicking the 10–23 DNAzyme substrate complex. This titration of the significantly larger and unlabeled DNAzyme snapback construct led to a supershift and enrichment of the representative dimer band (Fig. 3C lanes 3–6). The restriction enzyme digestion reports a higher proportion of 10–23 DNAzyme in the dimer conformation than was observed by EMSA. This is likely due to the constant removal of the dimer species from the reaction via restriction enzyme cleavage, which shifts the monomer/dimer equilibrium favoring dimerization. Together, both methods do confirm that the 10–23 DNAzyme is capable of dimerization in active conditions.

With the dimer accounting for ~ 5% of the DNAzyme population in solution, it is difficult to discern the contribution of this conformation to overall activity. In addition, the strict sequence requirements make mutagenesis difficult as the bases that are required for activity are bases that participate in dimer interaction, most notably the palindromic sequence. With the bases that participate in dimer coordination being necessary for activity and the low amount of dimer present in solution it is possible that the dimer could represent an auxiliary catalytic form to the monomeric DNAzyme. There have been previous kinetic studies indicating that the 10–23 DNAzyme does function as a monomer^22^. Since the dimer population is such a small percentage its contribution, if any, to the reaction rate is likely negligible.

### The substrate cleavage site is bent and exposed

**Figure 4:**
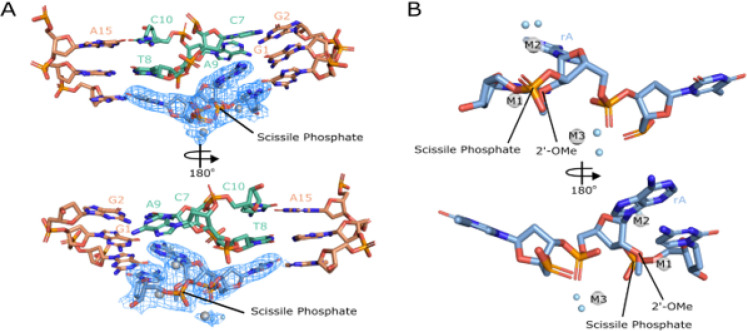
Organization of the active site. (A) Overview of active site with catalytic core bases in orange and paired catalytic core bases in green and substrate (blue) with electron density (s = 1.25) for consensus site bases. (B) Consensus site bases with metal ions coordinated (silver) labeled M1-M3.

**Figure 5:**
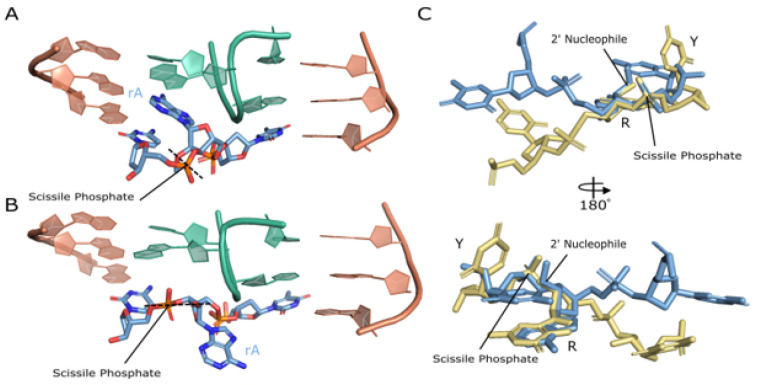
Active site comparisons. (A) active site of 10–23 DNAzyme crystal structure. (B) Proposed base-flip conformational change that aligns the 2’OH to the scissile phosphate for ‘in-line’ attack. (C) Alignment of the 10–23 DNAzyme crystal structure active site (blue) and the NMR 10–23 DNAzyme active site (yellow).

A hallmark of activity for RNA cleaving machines is bending the substrate to designate the cleavage site and position the scissile phosphate in a favorable geometry for cleavage to occur^23^. In this structure of the 10–23 DNAzyme, a bend is observed at the scissile phosphate and is coordinated through base pair interaction with the substrate binding arms at the − 1 and + 1 positions relative to the catalytic core and with G1 forming a non-canonical G-A base pair with the ribo-adenosine at the cleavage site. Additional contacts between the adjacent DNAzyme’s catalytic core stabilize this bend through stacking interactions with bases T8-C10 (Fig. 4A). The base stacking interactions between T8-C10 and the active site are due to the two regions of base pairing that coordinate the dimer structure of the 10–23 DNAzyme. The captured precatalytic active site conformation does not align the 2’OH nucleophile with the scissile phosphate in the pre-requisite in-line attack conformation often observed in RNA-cleaving machines^14,24^. Due to improper alignment of nucleophile to scissile phosphate it appears this active site is not in an active state and it is unclear if achieving an active state is possible for the dimer conformation. In order to align the nucleophile to the scissile phosphate a base flip of active site purine would be required (Fig. 5B), however, there is nothing within this structure to stabilize such a transition.

### Three magnesium ions are observed near the scissile phosphate

The bending of the substrate strand at the cleavage site allows for the coordination of 3 metal ions denoted M1, M2, M3 which has been previously suggested for the 10–23 DNAzyme^25^. Two magnesium ions are observed to coordinate to the 2’OH nucleophile, 3’ bridging oxygen, and the 5’ leaving group of the cleavage site. The additional metal ion is coordinated to a non-bridging oxygen on the scissile phosphate (Fig. 4B). To verify the identity of the metal ions in the structure, ytterbium soaks were trialed, but resulted in low resolution diffraction. However, each magnesium at the active site was also replaced with sodium and separately refined. (Supplemental Fig. 1). Upon replacement with sodium at each site M1-M3 a positive peak appeared in the Fo-Fc map surrounding the sodium atom indicating that additional electron density was required to satisfy the experimental observations. This suggests that all three metals coordinated to the active site are magnesium rather than sodium. While magnesium is a required cofactor for the 10–23 DNAzyme it is still uncertain how magnesium participates in the reaction. In the only monomeric structure of the 10–23 DNAzyme to date a catalytic core base G14 of the DNAzyme was observed to localize to the scissile phosphate to facilitate the cleavage mechanism with magnesium ions likely being involved in stabilizing this active conformation^18^. The magnesium coordination pattern observed in this crystal structure could largely be due to the availability of the scissile phosphate to solvent and the high concentration of magnesium that was required for crystallization to occur (200mM).

## Conclusions and Future Perspectives

Here we present a structure of the 10–23 DNAzyme that adopts a homodimer conformation with proper coordination of DNAzyme to substrate. While this dimer conformation is likely not catalytic in of itself it these misfolded conformations of the 10–23 DNAzyme are still present in solution. In addition, the recently solved monomeric structure of the 10–23 DNAzyme also did not showcase an active site with the 2’OH nucleophile properly organized to the scissile phosphate. Since only inactive forms of the 10–23 DNAzyme can be reliably observed, stable misfolded states of the catalytic core and improper organization of the active site may be key factors that slow reaction kinetics of the 10–23 DNAzyme.

Further structural work is required to verify these conclusions and to capture an in-line conformation of the 10–23 DNAzyme active site to observe what structural rearrangement occurs. Crystallography of the 10–23 DNAzyme has limitations in this respect as the dimer will be selected for due to the additional crystal contact and stability it provides. Therefore, the utilization of alternative methods will likely be needed to further elucidate the different 10–23 DNAzyme pre-catalytic conformations and in pursuit of capturing an in-line attack conformation. This structural data will be essential as the field continues to optimize the DNAzyme toward *in vivo* applications. Through the comparison of DNAzyme structures captured in the pre-, post-, and intermediate catalytic states it will be possible to identify the interactions that allow for proper cleavage site organization and activity. This could lead to the rationale design to stabilize the active conformation of the 10–23 DNAzyme and improve its overall catalytic efficiency^26^.

## Methods

### Oligonucleotide Synthesis

All 10–23 DNAzyme constructs used in this study for crystallization and for *in vitro* assays were purchased from IDT.

**Table:** 

Oligo Name	Sequence
10–23 Xstal mA	GCTGGGATmACATTGTGCGAAAGCACAATGGGCTAGCTACAACGAATCCCAGC
10–23 Xstal rA	GCTGGGArUrArCAATCCTAGTTATAGGATTGGGCTAGCTACAACGAATCCCAGC
FAM 10–23 DNAzyme	FAM-CTCGCAGTATAGGCTAGCTACAACGAATCGCATAGG
10–23 Substrate mA	CCTATGCGAUmACATACTGCGAG
10–23 SnapBack	TATCCGCGGAGACGCGGATAGGGCTAGCTACAACGAAGCCTATCGGAGACGATAGGC
10–23 DNAzyme	CTCGCAGTATAGGCTAGCTACAACGAATCGCATAGG
FAM 10–23 substrate	FAM-CCTATGCGArUrArCATACTGCGAG

### Protein Purification

Expression was performed from a pET11a vector containing a codon optimized synthetic Asfv Polx gene N-terminally tagged with His-SUMO (Genscript). Expression was conducted in *E. coli* Rosetta 2 (DE3) with LB autoinduction media containing 0.13 g/L MgSO_4_, 3.33 g/L (NH_4_)_2_SO_4_, 6.8 g/L KH_2_PO_4_, 7.1 g/L Na_2_HPO_4_, 0.5 g/L glucose, and 3.33 g/L lactose and incubated overnight at 25°C^27^. Cells were lysed via sonication in lysis buffer containing 20 mM Tris-HCl (pH7.5), 300 mM NaCl, 10 mM Imidazole and 5 mM Beta-mercaptoethanol (b-ME). The resulting lysate was cleared through centrifugation at 7068rcf (BIOFlex HC rotor; ThermoFisher) for 15 min and then at 29097rcf (F14–14×50cy rotor; ThermoFisher) for an additional 15 min. Ni-NTA resin (Gold Bio) was equilibrated into lysis buffer and introduced to the cleared lysate and allowed to bind for 30 min. The resin was then washed with buffer containing 20 mM Tris-HCl (pH7.5), 300 mM NaCl, 20 mM Imidazole, and 5 mM b-ME followed by an additional wash with high salt buffer (20 mM Tris-HCl (pH 7.5), 2M NaCl) before being eluted in buffer containing 20 mM Tris-HCl (pH7.5), 150 mM NaCl, 300 mM Imidazole, and 1 mM DTT. The subsequent elution was then treated with SUMO protease overnight at 4°C before being loaded onto a hi-Trap Q ion exchange column (GE) equilibrated into elution buffer from the Ni-NTA purification. Asfv Polx was eluted off the ion exchange with buffer containing 20 mM Tris-HCl (pH 7.5), 1.1 M NaCl, and 1 mM DTT before being concentrated to 13 mg/mL in a 10kDa MWCO filter (GE).

#### Crystallization:

10–23 DNAzyme crystallization oligo was resuspended to a concentration of 1 mM in buffer containing 20 mM Tris-HCl (pH 7.5), 150 mM NaCl, 50 mM MgCl_2_ and introduced to Asfv PolX (13mg/mL) in a 1:1 volumetric ratio with a final volume of 120 ml. 4 M NaCl was added to the sample at 1ul increments until the sample became free of any precipitation. 10–23 DNAzyme-Asfv PolX complex was screened by sitting drop vapor diffusion against Index screen (Hampton Research) at 22°C. Crystals formed in around 2 weeks. Final conditions were 0.2 M MgCl_2_, 0.1 M HEPES (pH 7.5), and 25% PEG-3350. Long rod crystals were cryoprotected using the crystallization solution supplemented with 30% ethylene glycol before being flash frozen in liquid nitrogen. Data acquisition was performed onNE-CAT24-IDE beam line at the Advanced Photon Source (APS) at Argonne National Laboratories.

### Structure Determination and Refinement

Data were indexed, integrated, and scaled using iMOSFLM and Aimless. The 10–23 DNAzyme structure was solved by molecular replacement (MR) with Asfv PolX (PDB 5XM8) using Phaser (Phenix software package). Model building/rebuilding was performed using Coot ^28^. Refinement was performed using Phenix ^29^.

#### Activity Assays:

5′-FAM labeled substrates and 10–23 DNAzyme activity constructs were purchased from IDT and resuspended in water. Reactions were performed with 100nM DNAzyme construct in 20 mM Tris-HCl (pH 7.5), 150 mM NaCl, and 25 mM MgCl_2_ at 37°C for 4hrs. Reactions were stopped by adding an equal volume of a 90% Formamide, 50 mM EDTA solution before heating at 90°C for 2 min and then immediately placed on ice for 3–5 min before separating on a 7 M urea, 20% acrylamide (19:1), 1X TBE denaturing PAGE gel. The resulting gel was imaged under a Cy2 filter on a Typhoon (GE) for FAM labeled products.

#### Native Electrophoretic Assays:

10–23 DNAzyme and substrate were introduced at 10:1 molar ratios of unlabeled:labeled with the FAM labeled reporter at a constant 100nM final concentration. The reactions were incubated in 20 mM Tris-HCl (pH7.5), 150 mM NaCl both with and without magnesium as specified. Glycerol was then added to 10% final glycerol concentration. Samples were separated on a 5% native polyacrylamide gel (28.5:1) containing 0.6X TB, 4% glycerol, 5 mM MgCl_2_ at 150 V for 3 hrs at 4°C. The resulting gel was imaged under a Cy2 filter on a Typhoon (GE) for FAM labeled products.

### Restriction Enzyme Assay

5′-FAM labeled 10–23 DNAzyme construct at 100nM was incubated both with and without 2′-OMe substrate (100nM) in 1X NEB cutsmart buffer, 20U Nhe I-HF (NEB) incubate at 37°C overnight. The reaction was then stopped and separated as described for the activity assay.
